# A case of Dressler’s syndrome after pulsed field ablation for atrial fibrillation

**DOI:** 10.1016/j.hrcr.2025.05.026

**Published:** 2025-06-03

**Authors:** Jaspal S. Gill, Mark M. Gallagher, Viral Sagar, Manav Sohal, Mark Specterman, Lisa W.M. Leung

**Affiliations:** Department of Cardiology, St. George’s Hospital NHS Foundation Trust, London, United Kingdom

**Keywords:** AF ablation, Persistent AF, Pulsed field ablation, Dressler’s, Pericarditis, Postcardiac injury syndrome

**Key Teaching Points**

• Pulsed field ablation (PFA) can cause postcardiac injury syndrome in a delayed setting similar to Dressler’s syndrome, suggesting that PFA may cause clinically relevant thermal effects and tissue necrosis.
• Thorough investigations including a computed tomography scan of the thorax with contrast and serial echocardiography remain important to rule out more extensive injury such as esophagopericardial fistulas.
• Medical management is similarly effective compared with treatment of Dressler’s syndrome owing to other causes.

## Introduction

Catheter ablation for atrial fibrillation (AF) is superior to pharmacologic rhythm control in certain patient populations, and there is evidence that early rhythm control improves patient outcomes and results in decreased disease progression.^1^ The safety of AF ablation should be the priority, with advances proposed in recent years ranging from refining the method and energy delivery of ablation to various device-related strategies to improve esophageal protection.

Pulsed field ablation (PFA) has been presented as a nonthermal method of ablation, with high-voltage electrical pulse waveform energy delivery being cardiomyocyte tissue selective. The mechanism of irreversible cellular membrane disruption is by electroporation leading to cellular apoptosis.^2^ This is in contrast to the tissue necrosis caused by radiofrequency and cryoablation energies. The cited safety advantages of PFA are tissue selectivity, sparing of the esophagus and phrenic nerve, and shorter left atrial dwell times.^2^ Owing to the rapid adoption of PFA since its introduction, operators are encouraged to report any PFA-related adverse events as experience is gained. To the best of our knowledge, this case report is the first to describe Dressler’s syndrome induced by PFA for AF ablation.

## Case report

A 55-year-old gentleman presented with symptomatic persistent AF and evidence of AF associated cardiomyopathy. He underwent elective direct current cardioversion 4 months after the clinical diagnosis and onset of symptoms. The left ventricular function normalized after restoration of sinus rhythm. Left atrial volume index via echocardiographic evaluation was 37 mL/m^2^ in sinus rhythm. The baseline electrocardiogram demonstrated a chronic right bundle branch block. He was listed for urgent elective AF ablation given the diagnosis of tachycardia-mediated cardiomyopathy.

He had a medical history of hypertension, hypercholesterolemia, nonalcoholic fatty liver, and a high body mass index (35.9 kg/m^2^; weight 113 kg). His medications were apixaban 5 mg twice daily, bisoprolol 1.25 mg once daily, atorvastatin 20 mg at night, ramipril 1.25 mg once daily, and spironolactone 12.5 mg once daily.

The patient attended for a planned ablation for persistent AF in November 2024. The procedure was performed under general anesthesia with transesophageal echocardiogram (TOE) guidance. The patient was in AF at the outset, and a direct current cardioversion was performed before mapping the left atrium under pacing from a decapolar catheter placed in the coronary sinus. A single transeptal puncture was performed using a Brockenbrough-1 needle (BRK-1, Abbott Medical, Chicago, IL) guided by fluoroscopy and TOE. Electroanatomic mapping was performed using the Orion multipolar catheter and Rhythmia mapping system (Boston Scientific, Marlborough, MA). There was normal pulmonary venous anatomy. Normal left atrial voltages were observed, and the volume was calculated at 156 mL. Pulmonary vein isolation was performed using PFA with a pentaspline catheter (Farawave, Boston Scientific). A total of 34 applications were administered. The standard 32 applications (4 each of basket and flower configurations to each vein) and 2 extra applications in flower configuration to the left-sided veins more anteriorly at the carina. Pulsed field lesions were delivered as per proprietary waveforms, with minimum time between lesions dictated by the proprietary Farapulse signal generator. Left atrial dwell time was 72 minutes and activated clotting time (ACT) target was 350 seconds. ACT values during left atrial dwell between 300 and 375 seconds were achieved, with no measurements significantly above the ACT target and a single measurement at 241 seconds. Acutely successful pulmonary vein isolation was confirmed by mapping with the Farawave catheter. There was no effusion visible on TOE at the end of the procedure, and no significant pericardial fat was identified. No acute complications were identified, and he was discharged home the next day in sinus rhythm.

Four days after ablation, the patient presented to the emergency department with pleuritic central chest pain without accompanying symptoms. Examination was unremarkable and the electrocardiogram showed sinus rhythm with right bundle branch block as per baseline. Blood tests showed a troponin T level of 252 ng/L (normal range <16), D-dimer of 69 ng/L (normal range 50–300), hemoglobin of 139 g/L (120–170), creatinine of 67 μmol/L (estimated glomerular filtration rate >90), and C-reactive protein (CRP) of 16 mg/L (normal range <5). Bedside echocardiography showed preserved biventricular function and trivial pericardial effusion. The troponin T level was downtrending from the value on discharge after ablation (281 ng/L). He was discharged from the emergency department with a presumed diagnosis of procedure-related pericarditis. The patient was prescribed a tapering course of colchicine and ibuprofen with a proton pump inhibitor for gastroprotection.

**Figure 1:**
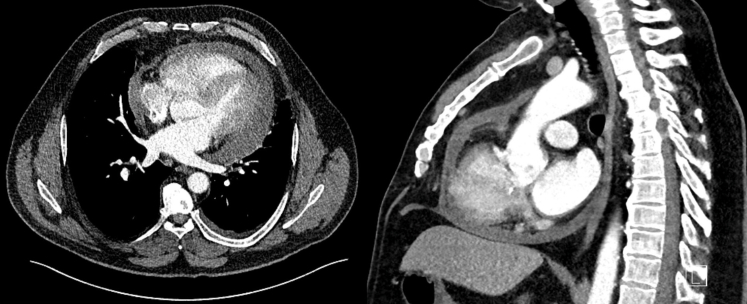
*Top:* Transverse plane image of contrast-enhanced computed tomography showing moderate circumferential pericardial effusion measuring 15–30 Hounsfield units. There is a small left-sided pleural effusion also seen. No evidence of contrast extravasation was demonstrated. *Bottom:* A sagittal plan image showing circumferential pericardial effusion, contrast in the left atrium, and no demonstrable communication with the esophagus.

At day 11 after ablation, the patient re-presented with symptoms of general malaise and fevers with rigors and night sweats. The CRP was now 138 mg/L. There was now a significantly increased circumferential pericardial effusion at 1.6 cm maximum depth without echocardiographic features of tamponade. He was admitted to the cardiology ward and the suspicion of esophagopericardial fistula prompted a cardiac gated computed tomography scan of the thorax (Figure 1). This revealed a moderate-sized pericardial effusion and a small left-sided pleural effusion but no evidence to support cardiac perforation or atrioesophageal fistula. Respiratory polymerase chain reaction swabs did not detect any pathogen. Urinalysis and microscopy were negative. Blood cultures were negative for any growth.

**Figure 2:**
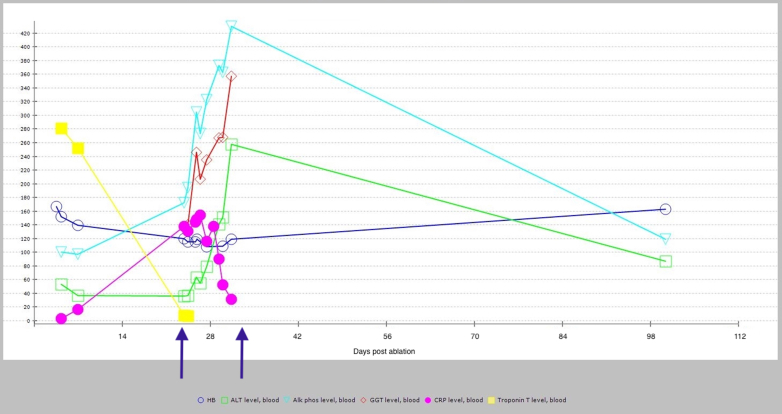
Blood result trends over time demonstrating acute change and normalization. The arrows indicate when the patient was admitted and discharged. Alk phos = alkaline phosphatase; ALT = alanine transaminase; CRP = C-reactive protein; GGT = gamma-glutamyl transferase; HB = hemoglobin.

Serial echocardiography over the subsequent days demonstrated a further increase in the size of the pericardial effusion to a maximum of 2.6 cm with features of increased tricuspid and mitral inflow Doppler respiratory variability but no evidence of overt chamber collapse (Figure 2).

**Figure 3:**
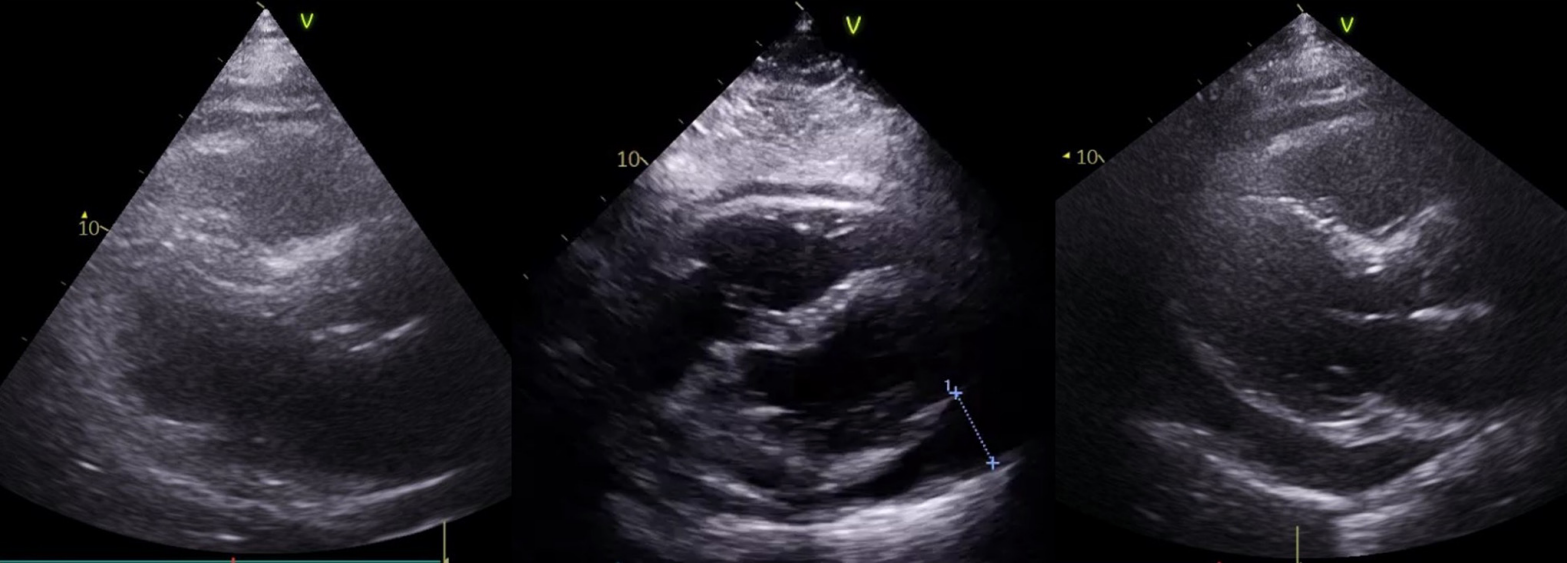
Transthoracic echocardiographic images showing progression of pericardial effusion. The leftmost image is a parasternal long-axis view at day 4. The middle image is a subcostal view at day 8. The rightmost image is a parasternal long-axis view at day 14 with circumferential pericardial effusion measured at a maximum of 2.6 cm. No significant pericardial fat was demonstrated on these echocardiographic images.

The patient was started on amoxicillin/clavulanic acid because of ongoing fevers, pleural effusion, and consequent possibility of a lower respiratory tract infection. A pericardiocentesis was considered but, given the lack of hemodynamic compromise or concerning echocardiographic features, it was deemed unnecessary. Blood cultures repeatedly found no growth. Troponin T levels were never elevated beyond the acute postablation period. The CRP remained elevated at 154 mg/L by day 12 after ablation when the patient’s presentation was treated as a postablation Dressler’s syndrome recalcitrant to nonsteroidal/colchicine therapy, and a switch was made to oral corticosteroid therapy with prednisolone 40 mg daily. Liver function tests were found to be transiently deranged (peak alanine transaminase 258 U/L, gamma-glutamyl transferase 357 U/L, alkaline phosphatase 430 U/L, bilirubin 5 μmol/L), and liver ultrasound demonstrated hepatic steatosis, as previously known (Figure 3). The transient derangement in liver function tests was attributed in part to amoxicillin/clavulanic acid antibiotics and also a systemic inflammatory response. Autoantibodies showed a weakly positive antinuclear antibody (1/80 speckled pattern), negative smooth muscle antibody, negative mitochondrial antibody, and negative double-stranded DNA. Immunoglobulins were recorded in the normal range and no paraprotein was detected on plasma electrophoresis. Alpha-1 antitrypsin levels were mildly elevated at 2.1 g/L (normal range 1.1–2.1 g/L), consistent with a systemic inflammatory response. Hepatitis A, B, and C serology were negative.

Corticosteroid therapy was met with the absence of further fevers, downtrending inflammatory markers to normal, and a significant decrease in effusion size when assessed with echocardiography. The patient was subsequently discharged on day 16 after ablation. A follow-up echocardiogram 2 weeks after discharge (day 30 after ablation) showed a trivial amount of pericardial fluid, and the CRP was normal. Corticosteroid therapy was weaned after 1 week of full dose by 10 mg per week over 4 weeks. He has been off all anti-inflammatory therapy since stopping corticosteroid therapy on day 47 after ablation. He now remains well and has maintained sinus rhythm off antiarrhythmic drugs since ablation. A further transthoracic echocardiogram performed 159 days after discharge showed no pericardial effusion.

## Discussion

To the best of our knowledge, this is the first recorded case of Dressler’s syndrome after PFA for the treatment of AF.

Postcardiac injury syndrome is a grouping of autoimmune-mediated responses to cardiac tissue necrosis and encompasses syndromes such as Dressler’s syndrome. Inflammation of the pericardium, myocardium, and pleura may be present, and they occur after a variable latency period after insult, on average 4–5 days. This inflammatory process typically occurs after myocardial infarction without revascularization, after pericardiotomy, or after trauma, reflecting a cascade of events from the initial ischemic tissue necrosis. In this patient, the clinical symptoms of fevers, sweats, and chest pain mounted >5 days after ablation, indicating a delayed onset, which would be in line with the definition of Dressler’s syndrome.

Dressler’s syndrome has been reported after radiofrequency, cryoballoon, or hybrid ablation for AF.^3^ Some data suggest that the more extensive the ablation performed, the higher the risk of postcardiac injury syndrome, although there are case reports of its occurrence in very limited ablation, suggesting that even focal cardiac tissue necrosis can cause this systemic reaction.^4^

In our case, there was a standard set of PFA applications with no procedural anomalies. It is theoretically plausible that the patient’s presentation could be related to procedure-related microperforation with slow bleeding into the pericardial space; however, the significantly raised inflammatory biomarkers observed and their prompt resolution with steroids are more suggestive of an inflammatory response. It had previously been believed that the lack of thermal effect of PFA and the mechanism of electroporation led to the process of apoptotic cell death rather than necrosis and that this led to a dampened inflammatory response to ablation.^5^ This case is significant because it supports the wider recognition that although PFA is considered to be relatively tissue specific, there remains the possibility of a thermal effect and therefore some degree of indiscriminate tissue necrosis. It is not yet known whether this means that the risk of esophageal injury still exists, although the data suggest the risk to be very low given that, to the best of our knowledge, there have been no reported cases in the largest series to date.^6^

There is a possibility that PFA could cause direct pericardial injury and thereby activate proinflammatory pathways related to pericardial fat. Activation or mediation of pericardial fat inflammation could occur owing to ablation of adjacent myocardial tissue and give rise to this patient’s presentation; however, there was an absence of significant pericardial fat identified on echo. Direct injury should cause a more acute or immediate inflammatory reaction but, in this case, it was delayed, similar to Dressler’s syndrome. Underpinning the exact pathophysiological mechanisms would be difficult without further clinical research or investigation of similar cases if or when they arise.

This case shows that Dressler’s syndrome can occur after AF ablation by PFA. This complication adds to the other reported PFA complications such as postprocedure neurologic events, acute cardiac tamponade, delayed phrenic nerve palsy, and hemolysis requiring renal support. This case underscores the need for more data or a detailed review to determine whether patient-specific or technology-specific factors, such as the number of applications, tissue contact, or waveform of the PFA system, could increase the risk of direct or indirect collateral injury.

## Disclosures

The authors have no conflicts of interest to disclose.

## References

[1] Andrade J.G. (2023). Ablation as first-line therapy for atrial fibrillation. Eur Cardiol.

[2] Jiang S., Qian F., Ji S. (2024). Pulsed field ablation for atrial fibrillation: mechanisms, advantages, and limitations. Rev Cardiovasc Med.

[3] Sasse T., Eriksson U. (2017). Post-cardiac injury syndrome: aetiology, diagnosis, and treatment. e-J Cardiol Pract.

[4] Wenzl F.A., Manninger M., Wunsch S., Scherr D., Bisping E.H. (2021). Post-cardiac injury syndrome triggered by radiofrequency ablation for AVNRT. BMC Cardiovasc Disord.

[5] Reddy V.Y., Anic A., Koruth J. (2020). Pulsed field ablation in patients with persistent atrial fibrillation. J Am Coll Cardiol.

[6] Ekanem E., Neuzil P., Reichlin T. (2024). Safety of pulsed field ablation in more than 17,000 patients with atrial fibrillation in the MANIFEST-17K study. Nat Med.

